# Active fractions of golden-flowered tea (*Camellia nitidissima Chi*) inhibit epidermal growth factor receptor mutated non-small cell lung cancer *via* multiple pathways and targets *in vitro* and *in vivo*

**DOI:** 10.3389/fnut.2022.1014414

**Published:** 2022-10-28

**Authors:** Ziling Wang, Xiaoying Hou, Min Li, Rongsheng Ji, Zhouyuan Li, Yuqiao Wang, Yujie Guo, Dahui Liu, Bisheng Huang, Hongzhi Du

**Affiliations:** ^1^Key Laboratory of Traditional Chinese Medicine Resources and Chemistry of Hubei Province, School of Pharmacy, Hubei University of Chinese Medicine, Wuhan, China; ^2^School of Medicine, Wuhan Institutes of Biomedical Sciences, Jianghan University, Wuhan, China; ^3^Shenzhen Luohu Hospital Group Luohu People’s Hospital, The Third Affiliated Hospital of Shenzhen University, Shenzhen, China

**Keywords:** golden-flowered tea, *Camellia nitidissima Chi* (CNC), non-small cell lung cancer (NSCLC), natural product, epidermal growth factor receptor (EGFR)

## Abstract

As a medicine-food homology (MFH) plant, golden-flowered tea (*Camellia nitidissima Chi*, CNC) has many different pharmacologic activities and is known as “the queen of the tea family” and “the Panda of the Plant world”. Several studies have revealed the pharmacologic effects of CNC crude extract, including anti-tumor, anti-oxidative and hepatoprotective activity. However, there are few studies on the anti-tumor active fractions and components of CNC, yet the underlying mechanism has not been investigated. Thus, we sought to verify the anti-non-small cell lung cancer (NSCLC) effects of four active fractions of CNC. Firstly, we determined the pharmacodynamic material basis of the four active fractions of CNC (*Camellia. leave. saponins*, *Camellia. leave. polyphenols*, *Camellia. flower. saponins*, *Camellia. flower. polyphenols*) by UPLC-Q-TOF-MS/MS and confirmed the differences in their specific compound contents. Then, MTT, colony formation assay and EdU incorporation assay confirmed that all fractions of CNC exhibit significant inhibitory on NSCLC, especially the *Camellia. leave. saponins* (CLS) fraction on EGFR mutated NSCLC cell lines. Moreover, transcriptome analysis revealed that the inhibition of NSCLC cell growth by CLS may be *via* three pathways, including “Cytokine-cytokine receptor interaction,” “PI3K-Akt signaling pathway” and “MAPK signaling pathway.” Subsequently, quantitative real-time PCR (RT-qPCR) and Western blot (WB) revealed TGFB2, INHBB, PIK3R3, ITGB8, TrkB and CACNA1D as the critical targets for the anti-tumor effects of CLS *in vitro*. Finally, the xenograft models confirmed that CLS treatment effectively suppressed tumor growth, and the key targets were also verified *in vivo*. These observations suggest that golden-flowered tea could be developed as a functional tea drink with anti-cancer ability, providing an essential molecular mechanism foundation for MFH medicine treating NSCLC.

## Introduction

Golden-flowered tea (*Camellia nitidissima Chi*—CNC) as an edible and medicinal plant (EMP) is an evergreen shrub belonging to the family Camellia (1). Golden-flowered tea has been known as “the panda of the plant world” and “the queen of the tea family” for its great ornamental and medicinal value. According to the “Guangxi Zhuang Autonomous Region Zhuang Medicine Quality Standard” (2), CNC has been used to treat various diseases such as pharyngitis, dysentery, liver cirrhosis and cancer for a long time. Most recently, CNC has been introduced and cultivated in Australia, Japan, the United States, and other countries (3). Moreover, a plethora of researchers are interested in the anti-cancer effects of CNC as a functional food.

Previous studies of CNC pharmacological effects had emphasized anti-tumor, anti-obesity and hypolipidemic effects (4, 5). In the last two decades, much of the research about CNC has explored the pharmacological effects of flower fractions, while the studies of leaf fractions are extremely rare. In fact, the leaves of CNC have been used as tea for a long time (6). Although, several studies have revealed the anti-cancer effects of CNC leaves crude extract, there are few reports on the anti-tumor active fractions and components of CNC (7). Thus, it is necessary to explore the differences in the pharmacological effects of different active fractions of CNC.

Previously, our research for the first time confirmed that the four active fractions of CNC (*Camellia. leave. saponins, Camellia. leave. polyphenols, Camellia. flower. saponins, Camellia. flower. polyphenols*) effectively inhibited the proliferation, metastasis and invasion of anti-NSCLC *in vitro* (8), while the anti-cancer mechanism remains to be revealed. Lung cancer is the leading type of cancer death worldwide, with NSCLC being the most common sub-type (9–12), accounting for approximately 85% (13). Among the emerging oncology therapies, molecular targeted drugs have become the first choice for treating NSCLC (14). Approximately 10–40% of NSCLC patients worldwide have tumor cells carrying epidermal growth factor receptor (EGFR) activating mutations (15). The epidermal growth factor receptor-tyrosine kinase inhibits (EGFR-TKI) targeted therapy is a milestone in tumor treatment with remarkable effects (16). However, NSCLC frequently develops acquired resistance when treated with NSCLC owing to factors such as tumor mutational burden (17), immune evasion and tumor microenvironment (TME) (18, 19). Therefore, the search for new therapeutic agents for drug-resistant NSCLC and the analysis of medicinal treatment mechanisms are frontier issues in oncology science, which have scientific value and clinical guidance significance for the treatment of NSCLC. Thus, we attempt to explore the molecular mechanism to provide more scientific evidence for the application of golden-flowered tea in the treatment of NSCLC.

**FIGURE 1:**
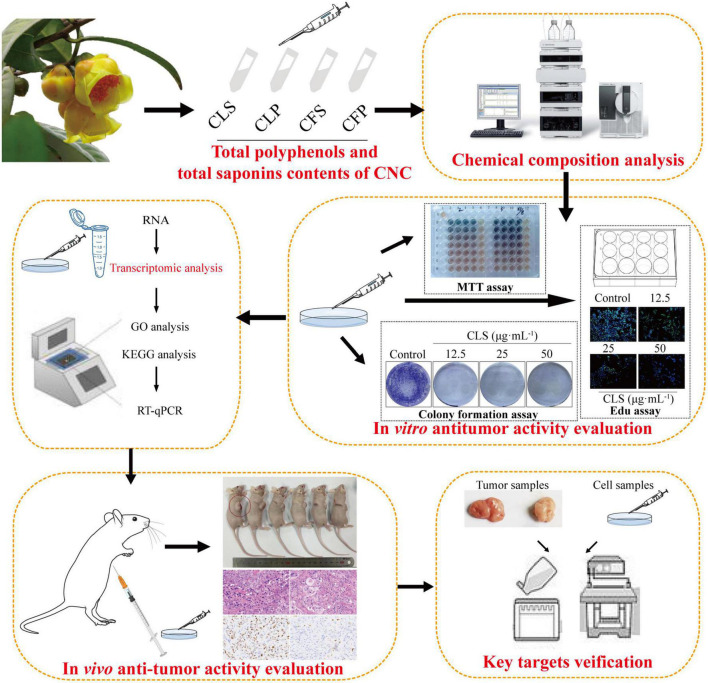
Experimental design ideas.

In brief, the component difference between the four fractions of CNC was first reported in this study. Then, we evaluated the anti-tumor activity of four fractions of CNC on three different NSCLC cell lines. To determine the programmed cell death effect on non-small cell lung cancer cells, we investigated whether CLS treatment induces the apoptosis of NCI-H1975 cells by TdT-mediated dUTP Nick-End Labeling (TUNEL) assay, Annexin V and propidine iodide (PI) staining, reactive oxygen species (ROS) measurement, superoxide dismutase (SOD) measurement, SEM examination and lactate dehydrogenase (LDH) release. Transcriptomics analysis was employed to probe the genetic changes after treatment of NSCLC cells with CLS. Subsequently, RT-qPCR and WB confirmation were performed for the candidate pathways. Finally, Xenograft models assay also proved the inhibitory effect of CLS *in vivo*. Taken together, our study investigated the inhibitory effect of different fractions of CNC on NSCLC (Figure 1). Importantly, our work will facilitate the study of the anti-tumor effect and mechanism of CNC as a functional tea.

## Material and methods

### Extraction of chemical constituents

The leaves and flowers of CNC were collected from Fangchenggang, Guangxi Province, China. The extraction method was based on the previous research of our team (7, 20). The leaves and flowers were air-dried and grinded into powder. The powder of leaves (6.3 kg) and flowers (6.0 kg) were separately refluxed with 95% ethanol for 3 times (3, 2 and 1 h). The extracts were combined and evaporated in a rotary evaporator to obtain ethanol extracts. Finally, four different active fractions of CNC (*Camellia. leave. polyphenols, Camellia. flower. polyphenols, Camellia. leave. saponins, Camellia. flower. saponins*) were obtained by macroporous resin purification process (21, 22).

### Chemical characterization of active fractions of *Camellia nitidissima Chi*

#### Determination of total polyphenols

Total polyphenols were determined by Follin-Ciocalteu (FC) assay. The FC reagent (diluted 1:10 in water) and aqueous Na2CO3 (10%) were added to the two fractions of CNC (*Camellia. leave. polyphenols, Camellia. flower. polyphenols*) in sequence. Gallic acid control solution was prepared to draw the calibration curve. The absorbance value was measured at 765 nm after constant shaking at 37°C for 30 min.

#### Determination of total saponins

Total saponins were determined by Vanillin-acetate method. Firstly, 5.0 g of vanillin was weighed to configure a 5% solution of vanillin acetate. Ginsenoside Re control solution was prepared to draw the calibration curve. Prepared 1 mL of 1 mg/mL of the solution (*Camellia. leave. saponins, Camellia. flower. saponins*) to be measured in the test tube in a water bath to evaporate. The 5% vanillin-acetate solution and Perchloric acid were added to the two fractions of CNC in sequence. After heating the test tubes at 60 degrees for 10 min, the test tubes were cooled with ice water and 5 mL of glacial acetic acid was added. The absorbance value was measured at 560 nm.

### Qualitative and quantitative analysis of active fractions of *Camellia nitidissima Chi*

The four active fractions of CNC were identified by the UPLC-Q-TOF-MS/MS. After being dissolved in methanol, the sample was filtered through a 0.22 μm microfiltration membrane for analysis. The UPLC-Q-TOF-MS/MS has equipped with an Agilent SB-C18 (1.8 μm, 2.1 mm × 100 mm) column. The mobile phase is composed of solvent A, 0.1% formic acid in water and solvent B, 0.1% formic acid of acetonitrile. The elation gradient procedure was performed: 0–9 min, 5–95% B; 9–10 min 95% B; 10–11.10 min 95–5% B; 11.10–14 min 5% B. The flow rate was 0.35 mL/min and the sample injection volume was 4 μL. The effluent was alternatively connected to an ESI-triple quadrupole-linear ion trap (QTRAP)-MS. The ESI source operation parameters were as follows: an ion source, turbo spray; source temperature 550°C; ion spray voltage (IS) 5,500 V (positive ion mode)/-4,500 V (negative ion mode); ion source gas I (GSI), gas II (GSII), curtain gas (CUR) was set at 50, 60, and 25.0 psi, respectively; the collision-activated dissociation (CAD) was high (23).

### Cell culture

NCI-H1975 cells, A549 cells and HCC827 cells were grown in RPMI Medium 1,640 basic (1×) supplemented with 10% fetal bovine serum(GEMINI BIO-Products)in a humidified chamber with 5% CO_2_ and 37°C. The cell culture method is the same as the previous culture method of our team (24).

### Cell viability assay

NCI-H1975 cells, A549 cells and HCC827 cells were seeded at 3,000, 5,000, and 3,000 cells per well of 96-well plates in triplicate. Cell viability was measured at 72 h by using a 3-(4, 5-dimethylthiazol-2-yl)-2, 5-diphenyl tetrazolium bromide (MTT) assay.

### Colony formation assay

NCI-H1975 and HCC827 cells were seeded at 500 cells per well of 6-well plates in a medium containing Penicillin-Streptomycin-Gentamicin Solution (Solarbio, P1410). After 24 h of incubation, the cells were treated with different concentrations of CNC every 5 days until colonies formed in 10 days. The remaining colonies were stained with crystal violet.

### EdU incorporation assay

After NCI-H1975 and HCC827 cells were treated by different fractions of CNC in 12-well plates for 24 h, the cells were cultured with 10 μM EdU (KevGEN BioTECH, KGA331-500) for 2 h, followed by incubation with 4% polychloraldehyde for 15 min. Washed by 3% BSA in PBS twice, the cells were incubated with 0.5% Triton X-100 (Solarbio, 9002-39-1) in PBS for 20 min. The cell plates were washed twice with 3% BSA in PBS and incubated with a 1 × Click-iT reactant mixture for 30 min. The cells treated with 1 × Click-iT reactant mixture were incubated with 1 × Hochest 33342 for 30 min under dark conditions. The proliferating cells (green) and the nuclei of all cells were observed under a laser confocal microscope under dark conditions. Different visual fields were randomly taken for image collection and synthesis analysis. Finally, the proliferation rate was calculated.

### TdT-mediated dUTP Nick-End Labeling staining

After NCI-H1975 were cultured in 12-well plates for 24 h, the cells were treated with different concentration of CLS for 24 h. Cells were subsequently incubation with 4% polychloraldehyde for 30 min. Washed by PBS twice, the cells were incubated with 0.3% Triton X-100 (Solarbio, 9002-39-1) in PBS for 10 min. The cell plates were washed twice with PBS and incubated with a TUNEL reactant mixture for 60 min at 37°C (Beyotime, C1086). Different visual fields were randomly taken for image collection and synthesis analysis. And the TUNEL positive rate was calculated and normalized to that of the control group.

### Annexin V and propidine iodide staining

The apoptosis rate of NCI-H1975 cells using Annexinv-fluorescein isothiocyanate (FITC) and PI double staining technique (KevGEN BioTECH, KGA107). NCI-H1975 cells were processed at different concentrations of CLS for 48 h. The cells were collected by digestion with EDTA-free trypsin and washed twice with PBS. After processing according to the steps in the instructions, all groups were measured by flow cytometer.

### Reactive oxygen species measurement

The effect of different concentrations on CLS-mediated ROS production in NCI-H1975 cells was determined using the cell-permeable fluorescent probe 2’,7’-dihydrofluorescein-diacetate (DCFH_2_-DA). NCI-H1975 cells were incubated in different concentration of CLS for 24 h, the cells were cultured with 1 μM DCFH_2_-DA (Solarbio, D6470) for 30 min at 37°C. And the ROS positive rate was calculated and normalized to that of the control group.

### Superoxide dismutase measurement

The effect of different concentrations on CLS-mediated SOD production in NCI-H1975 cells was determined using the SOD reagent kit (Njjcbio, A001-3-2). After being processed at different concentrations of CLS for 48 h, the proteins of NCI-H1975 cells were extracted. The relative content of SOD was determined by the reagent kit. The SOD positive rate was calculated and normalized to the control group.

### Scanning electron microscope examination

After NCI-H1975 cells were treated in accordance with the above-described experimental design, SEM was used to observe the difference between the treated and control groups. After cell crawling was washed with PBS, electron microscope fixative (Servicebio, G1102) was added and placed in a four-degree refrigerator for 1 h. Ethanol gradients were used to remove water from the samples, with dehydrating agent concentrations of 30, 50, 70, 80, 90, and 100% (twice) in order, with each dehydration time of 5 min. Finally, the samples were dried in the desiccator for 1.5 h and then sprayed with gold and photographed.

### Lactate dehydrogenase release

The release of IL-1β and LDH can be detected during the onset of pyroptosis (25). After NCI-H1975 cells were treated in accordance with the above-described experimental design, LDH release was measured by LDH assay kit (Njjcbio, A020-2) to observe the difference between the treated and control groups. The absorbance was measured at a wavelength 450 nm using microplate reader.

### Ribonucleic acid sequencing analysis and differential expression analysis

RNA degradation and contamination were monitored on 1% agarose gels. The preparation of each RNA sample requires 3 μg of RNA as input material (26). Sequencing libraries were generated using the NEBNext^®^ Ultra^TM ^®^^ RNA Library Preparation Kit (NEB, USA), and index codes were added to the attribute sequences of each sample. After the library inspection is qualified, the different libraries are pooled according to the requirements of effective concentration and target data volume. And illumine sequencing is performed, and the generated 150 bp paired-end reads. Differential expression analysis was performed for two conditions/groups (two biological replicates per condition) using the DESeq2 R package (1.16.1). Genes identified by DESeq2 with adjusted *p-*values < 0.05 were designated as differentially expressed genes. Gene Ontology (GO) enrichment analysis and Kyoto Encyclopedia of Genes and Genomes (KEGG) analysis of differentially expressed genes was implemented by the cluster profile R package (27, 28).

### Reverse transcription and real time-quantitative polymerase chain reaction

Total RNA was extracted from cell culture samples using the TRNzol Universal Reagent (Tiangen, W9712) according to the manufacturer’s instructions. The cDNA was synthesized from total RNA (1 μg) using reverse transcription (Vazyme, R323-01). Primer sequences were as follows in Table 1. PCR amplification was executed by the SYBR Green PCR master mix (LightCycler 480, 30408), and the PCR-amplified gene products were analyzed.

**TABLE 1:** Primer sequences.

Gene	Forward sequence	Reverse sequence
18S	AGGTCTGTGATGCCCTTAGATG	TCCTCGTTCATGGGGAATAATTG
INHBB	GAAATCATCAGCTTCGCCGAGAC	GGCAGGAGTTTCAGGTAAAGCC
TGFB2	AAGAAGCGTGCTTTGGATGCGG	ATGCTCCAGCACAGAAGTTGGC
PIK3R3	CCACCTAAGCCAATGACTTCAGC	GTTGAGGCATCTCGGACCAAGA
ITGB8	CTGTTTGCAGTGGTCGAGGAGT	TGCCTGCTTCACACTCTCCATG
TNFRSF10C	GGTGTGGATTACACCAACGCTTC	CTGACACACTGTGTCTCTGGTC
MEF2C	TCCACCAGGCAGCAAGAATACG	GGAGTTGCTACGGAAACCACTG
EIF4E1B	GACAAGATCGCTGTGTGGACGA	GTTGCTCTTGGTGGCTGTGTCT
TrkB	ACAGTCAGCTCAAGCCAGACAC	GTCCTGCTCAGGACAGAGGTTA
CACNA1D	CTTCGACAACGTCCTCTCTGCT	GCCGATGTTCTCTCCATTCGAG
IL-1β	TGCTCAAGTGTCTGAAGCAG	TGGTGGTCGGAGATTCGTAG

### Western blot analysis

After the cells were treated with CLS for the indicated time, cell lysates were lysed by RIPA buffer supplemented with a complete protease and phosphatase inhibitor mixture (Beyotime, 45482). Samples of mouse tumor tissues were stored in a −80°C refrigerator and homogenized with RIPA (Solarbio, 676). Proteins were separated on a 6–10% SDS-PAGE system and transferred to a polyvinylidene fluoride (PVDF) membrane. WB was performed according standard protocol with following primary antibodies: Anti-ACTB (Abclonal, AC004; 1:10,000), Anti-INHBB (Abclonal, A8553;1:1,000), Anti-TGFB2 (Abclonal, A3640; 1:1,000), Anti-ITGB8 (Abclonal, A8433; 1:1,000), Anti-PIK3R3 (Abclonal, A17112; 1:1,000), Anti-TrkB (Abclonal, A2099; 1:1,000), Anti-CACNA1D (Abclonal, A16785; 1:1,000), HPR Goat Anti-Mouse (Abclonal, AS003; 1:2,000) or HPR Goat Anti-Rabbit (Abclonal, AS014; 1:2,000) secondary antibodies were used. Protein bands were visualized by chemiluminescence reagents (Meilunbio, MA0186-1) and were performed using a luminescent image analyzer (Proteinsimple, 601577). Raw data were analyzed by using Fuji film v3.0.

### Xenograft models

Thirty 5-week-old BALB/c-nude mice were obtained from the Biont (Jiangsu, No.320727210100432325). All mice were housed in a temperature-controlled environment (24 ± 2°C) with a 12/12 h dark/light cycle at the Animal Center of Hubei university of Chinese medicine. The standard rat chow and water used for animal feeding and all animal experiments were conducted by the animal ethics-related regulations of Hubei University of Traditional Chinese Medicine, permission number: SYXK2017-0067-ZYZYZX2022-2. BALB/c-nude mice were injected subcutaneously in the armpit with NCI-H1975 (5 × 10^6^ cells/mice) in 150 μL PBS. After the mean tumor volume reached 50 mm^3^, BALB/c-nude mice were randomly divided into model control group (*n* = 6), tax group (*n* = 6), low-dose group (*n* = 6), medium-dose group (*n* = 6), high-dose group (*n* = 6). Low-dose orally took 100 mg/kg CLS every day, medium-dose orally took 200 mg/kg CLS every day, and high-dose orally took 400 mg/kg CLS every day. And taxol (anhydrous ethanol: castor oil = 1:1) was injected at 20 mg/kg every 2 days in the tail vein. Tumor volume was monitored by vernier calipers throughout the experiment. All mice were executed and tumors were removed on day 13.

$R\,T\,V=\frac{V\,t}{V\,0}$, where V0 represents the tumor volume of day 1 (the day of CLS first administration), Vt represents the tumor volume of day 13 (29).

### Immunohistochemistry

Samples from the tumor xenografts and liver were dissected, formalin-fixed and paraffin-embedded. Paraffin blocks were placed on the pre-cooling table and adjusted the knees to 4-M m thickness. Sections were incubated with citric acid (pH 6.0) antigen retrieval buffer (Beyotime, P0085) for antigen retrieval in a microwave oven. After blocking endogenous peroxidase with 3% hydrogen peroxide, and serum sealing by 3% BSA (Beyotime, P0007), sections were then incubated by Ki67 antibody (Abcam, Ab16667) and further processed with secondary antibody (Abcam, Ab6721). The chromogenic reaction was performed by DAB (Solarbio, DA1010). Sections were counterstained with hematoxylin (Solarbio, H8070) and observed under a microscope. The nucleus of hematoxylin stained is blue, and the positive expression of DAB is brownish yellow.

### Data presentation and statistical analysis

All graphs were generated using GraphPad Prism 8.0 (SanDiego, CA, USA). One-way ANOVA with Bonferroni correction was used for statistical analyses. Statistical significance was set at *p < 0.05, and **p < 0.01 compared to control unless stated differently.

## Results

### Total polyphenols and total saponins contents of *Camellia nitidissima Chi*

As is known, total polyphenols and total saponins are important active ingredients in tea beverages. Therefore, we first determined the contents of total polyphenols and total saponins in each of the four active fractions of CNC. The polyphenols contents of *Camellia. leave. polyphenols* (CLP) and *Camellia. flower. Polyphenols* (CFP) in terms of gallic acid equivalent (standard curve equation: y = 4.2285x+0.0597, *r*^2^ = 0.999) were from 20 to 100 μg/mL and listed in Table 2. The polyphenols contents in CLP were 136.89 ± 3.18 mg/g and the phenolic contents in CFP were 327.03 ± 4.03 mg/g. Table 2 also showed the content of total saponins reported as Ginsenoside Re equivalent (standard curve equation: y = 1.4807x+0.0223, *r*^2^ = 0.99), which were from 0.02 to 0.14 mg/mL. Saponin contents were 38.83 ± 0.57 mg/g in CLS and 56.53 ± 0.83 mg/g in CFS as shown in Table 2. The results indicated that total saponins and total polyphenols might be important active components in goldenrod tea.

**TABLE 2:** Total polyphenols and saponins in different fractions of CNC (*n* = 6).

Bio active substance	Fraction of CNC	mg/g dry mass
Total polyphenol content	CLP	136.89 ±3.18
	CFP	327.03 ±4.03
Total saponin content	CLS	38.83 ±0.57
	CFS	56.53 ±0.83

### Active compounds analysis in *Camellia nitidissima Chi* by UPLC-QTOF-MS/MS

**FIGURE 2:**
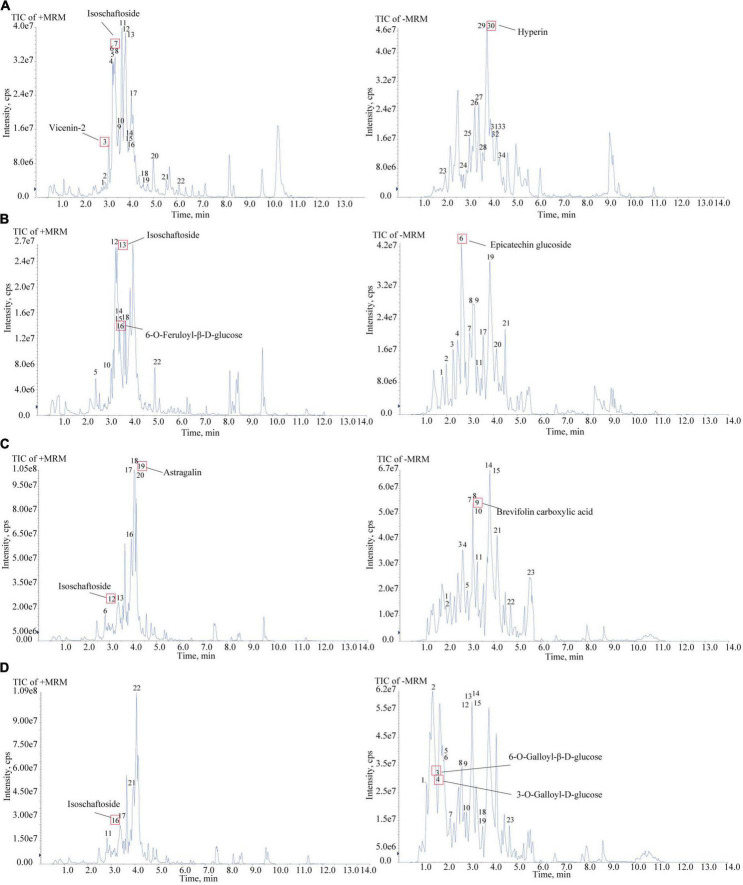
Chemical composition analysis of CLS, CLP, CFS, and CFP by using UPLC-QTOF-MS. **(A)** Total Ion Chromatography (TIC) in the positive and negative ion mode of CLS by UPLC-QTOF-MS. **(B)** Total Ion Chromatography (TIC) in the positive and negative ion mode of CLP by UPLC-QTOF-MS. **(C)** Total Ion Chromatography (TIC) in the positive and negative ion mode of CFS by UPLC-QTOF-MS. **(D)** Total Ion Chromatography (TIC) in the positive and negative ion mode of CFP by UPLC-QTOF-MS/MS.

To confirm the material basis of CNC, the active chemical components of the four active fractions of CNC were detected separately by UPLC-QTOF-MS/MS. As is shown in Figure 2A and Table 3, the three components with the highest content in CLS are isoschaftoside, hyperin and vicenin-2. In CLP, the three main effective compounds are 6-O-Feruloyl-β-D-glucose, epicatechin glucoside and isoschaftoside (Figure 2B and Table 3). However, astragalin, isoschaftoside and brevifolin carboxylic acid are the most abundant substances in CFS (Figure 2C and Table 4). And in CFP, 6-O-Galloyl-β-D-glucose, 3-O-Galloyl-D-glucose and isoschaftoside demonstrated extremely high content (Figure 2D and Table 4). In conclusion, we initially revealed the specific chemical composition of different fractions of CNC, which laid the foundation for the subsequent activity study.

**TABLE 3:** Compounds of CLS and CLP.

Fractions of CNC	Compound	Rt (min)	Molecular formula	Ion	Tentative identification	Measured m/z	MS/MS	Relative content (%)
CLS	1	2.94	C_33_H_40_O_21_	[M+H]^+^	Quercetin-3-O-rutinoside-7-O-glucoside	773.21	303.05	0.003
	2	2.97	C_15_H_14_O_6_	[M+H]^+^	Catechin	291.09	139.04	0.09
	3	3.11	C_27_H_30_O_15_	[M+H]^+^	Apigenin-6,8-di-C-glucoside (vicenin-2)*	595.17	457.20	2.36
	4	3.28	C_15_H_14_O_6_	[M+H]^+^	Epicatechin	291.09	139.04	0.83
	5	3.32	C_17_H_24_O_10_	[M+H]^+^	Geniposide	389.14	209.08	1.89
	6	3.34	C_26_H_28_O_14_	[M+H]^+^	Schaftoside	565.16	529.13	0.02
	7	3.35	C_26_H_28_O_14_	[M+H]^+^	Isoschaftoside*	565.16	409	4.39
	8	3.38	C_16_H_22_O_9_	[M+H]^+^	Swerosideit	359.13	197	2.1
	9	3.51	C_22_H_18_O_11_	[M+H]^+^	Gallocatechin gallate	459.09	139.04	0.006
	10	3.52	C_21_H_20_O_13_	[M+H]^+^	Myricetin-3-O-glucoside	481.10	319.04	0.008
	11	3.66	C_27_H_30_O_16_	[M+H]^+^	Quercetin-3-O-glucoside-7-O-rhamnoside	611.16	303.05	1.01
	12	3.77	C_33_H_40_O_20_	[M+H]^+^	Kaempferol-3-O-(6”-Rhamnosyl-2”-Glucosyl) Glucoside (camelliaside A)	757.22	287.06	1.36
	13	3.77	C_21_H_20_O_10_	[M+H]^+^	Apigenin-8-C-Glucoside (vitexin)	433.11	313.07	0.46
	14	3.83	C_21_H_20_O_12_	[M+H]^+^	Quercetin-5-O-β-D-glucoside	465.10	303	2.08
	15	3.90	C_27_H_30_O_15_	[M+H]^+^	Kaempferol-3-O-glucorhamnoside	595.17	287.06	0.29
	16	4.00	C_20_H_18_O_11_	[M+H]^+^	Quercetin-3-O-xyloside (reynoutrin)	435.09	303.06	0.35
	17	4.04	C_30_H_26_O_12_	[M+H]^+^	Apigenin-7-O-(6”-p-Coumaryl) glucoside	579.15	271	0.89
	18	4.16	C_21_H_20_O_10_	[M+H]^+^	Apigenin-7-O-glucoside (cosmosiin)*	433.11	271	0.34
	19	4.34	C_21_H_20_O_11_	[M+H]^+^	Quercetin-3-O-rhamnoside (quercitrin)	449.11	303.05	0.02
	20	5.02	C_15_H_10_O_7_	[M+H]^+^	Quercetin	303.05	137	1.82
	21	5.67	C_15_H_10_O_6_	[M+H]^+^	Kaempferol	287.06	153.02	0.14
	22	6.12	C_11_H_12_O_3_	[M+H]^+^	p-Coumaric acid ethyl ester	193.09	147	0.68
	23	1.74	C_7_H_6_O_5_	[M-H]^–^	Gallic acid	169.01	125	0.68
	24	3.05	C_7_H_6_O_3_	[M-H]^–^	Salicylic acid	137.03	108	0.26
	25	3.08	C_7_H_6_O_3_	[M-H]^–^	Protocatechualdehyde	137.02	93	0.45
	26	3.40	C_9_H_10_O_2_	[M-H]^–^	p-Coumaryl alcohol	149.06	131	0.002
	27	3.75	C_21_H_20_O_10_	[M-H]^–^	Apigenin-6-C-glucoside (isovitexin)	431.10	311	1.2
	28	3.76	C_36_H_36_O_18_	[M-H]^–^	Kaempferol-3-p-coumaroyldiglucoside	755.19	285	0.1
	29	3.81	C_21_H_20_O_12_	[M-H]^–^	Quercetin-3-O-glucoside (isoquercitrin)	463.09	300	2.08
	30	3.81	C_21_H_20_O_12_	[M-H]^–^	Quercetin-3-O-galactoside (hyperin)*	463.09	300	2.45
	31	3.84	C_22_H_18_O_10_	[M-H]^–^	Epicatechin gallate*	441.08	169	0.03
	32	3.84	C_22_H_18_O_10_	[M-H]^–^	Catechin gallate*	441.08	169.02	0.03
	33	4.21	C_15_H_10_O_7_	[M-H]^–^	Morin	301.04	151	1.9
	34	5.87	C_30_H_46_O_4_	[M-H]^–^	Camaldulenic acid	469.33	425	1.92
CLP	1	1.86	C_13_H_18_O_8_	[M-H]^–^	4-O-Glucosyl-3,4-dihydroxybenzyl alcohol	301.09	139.05	1.36
	2	1.94	C_13_H_16_O_9_	[M-H]^–^	Protocatechuic acid-4-O-glucoside	315.07	153.02	1.35
	3	2.29	C_14_H_20_O_9_	[M-H]^–^	2-(3,4-dihydroxyphenyl) ethanediol 1-O-β-D-glucopyranoside	333.1	153.02	2.21
	4	2.44	C_21_H_24_O_11_	[M-H]^–^	Epicatechin-4’-O-β-D-glucopyranoside	451.13	289.0	0.96
	5	2.47	C_11_H_12_N_2_O_2_	[M+H]^+^	1-Methoxy-indole-3-acetamide	205.1	146.06	1.01
	6	2.63	C_21_H_24_O_11_	[M-H]^–^	Epicatechin glucoside*	451.12	289.07	3.43
	7	2.97	C_15_H_18_O_8_	[M-H]^–^	p-Coumaric acid-4-O-glucosidep-Coumaric acid-4-O-glucoside	325.09	163.04	2.36
	8	3.1	C_7_H_6_O_3_	[M-H]^–^	Protocatechualdehyde	137.02	93.04	0.99
	9	3.11	C_7_H_6_O_3_	[M-H]^–^	4-Hydroxybenzoic acid	137.02	93	1.52
	10	3.14	C_27_H_30_O_15_	[M+H]^+^	Apigenin-6,8-di-C-glucoside (vicenin-2)	595.17	457.2	1.01
	11	3.18	C_16_H_20_O_9_	[M-H]^–^	1-O-Feruloyl-β-D-glucose	355.1	193.05	2.42
	12	3.32	C_17_H_24_O_10_	[M+H]^+^	Geniposide	389.14	209.08	1.68
	13	3.35	C_26_H_28_O_14_	[M+H]^+^	Isoschaftoside*	565.16	409.09	2.89
	14	3.38	C_16_H_22_O_9_	[M+H]^+^	Sweroside	359.13	197	1.36
	15	3.38	C_26_H_28_O_14_	[M+H]^+^	Apigenin-6-C-(2”-glucosyl) arabinoside	565.16	427.1	1.83
	16	3.44	C_29_H_39_N_3_O_8_	[M+H]^+^	N1, N8-Bis (sinapoyl) spermidine	558.28	207.07	1.95
	17	3.57	C_16_H_20_O_9_	[M-H]^–^	6-O-Feruloyl-β-D-glucose*	355.1	193.05	3.85
	18	3.67	C_27_H_30_O_14_	[M+H]^+^	Isovitexin-2’-O-rhamnoside	579.17	313.07	1.00
	19	3.88	C_9_H_10_O_5_	[M-H]^–^	Gallic acid ethyl ester	197.05	124.02	1.63
	20	3.89	C_27_H_30_O1_5_	[M-H]^–^	Kaempferol-3-O-robinobioside (biorobin)	593.15	285	1.08
	21	4.5	C_15_H_8_O_8_	[M-H]^–^	3-O-Methylellagic acid	315.01	299.99	0.99
	22	4.98	C_15_H_10_O_7_	[M+H]^+^	Quercetin	303.05	137.02	1.09

*Indicates the top three compounds from different fractions of CNC with the highest relative content.

**TABLE 4:** Compounds of CFS and CFP.

Fractions of CNC	Compound	Rt (min)	Molecular formula	Ion	Tentative identification	Measured m/z	MS/MS	Relative content (%)
CFS	1	1.94	C_14_H_20_O_9_	[M-H]^–^	Vanilloloside	315.11	153.02	1.33
	2	1.94	C_13_H_16_O_9_	[M-H]^–^	Protocatechuic acid-4-O-glucoside	315.07	153.02	1.55
	3	2.68	C_15_H_8_O_9_	[M-H]^–^	Vanillic Acid-4-O-Glucuronide	341.09	161.02	1.38
	4	2.68	C_15_H_8_O_9_	[M-H]^–^	1-O-Caffeoyl-β-D-glucose	341.09	161.02	1.32
	5	2.75	C_20_H_20_O_14_	[M-H]^–^	1,4-Di-O-Galloyl-D-glucose	483.08	169.01	1.73
	6	2.83	C_27_H_30_O_17_	[M+H]^+^	Quercetin-3,7-Di-O-glucoside	627.14	303.05	1.23
	7	3.10	C_7_H_6_O_3_	[M-H]^–^	Protocatechualdehyde	137.02	93.04	1.13
	8	3.10	C_30_H_26_O_12_	[M-H]^–^	Procyanidin B2	577.14	407.08	1.02
	9	3.11	C_13_H_8_O_8_	[M-H]^–^	Brevifolin carboxylic acid*	291.01	247	2.15
	10	3.11	C_7_H_6_O_3_	[M-H]^–^	4-Hydroxybenzoic acid	137.02	93	1.60
	11	3.29	C_14_H_10_O_7_	[M-H]^–^	4-(3,4,5-Trihydroxybenzoxy) benzoic acid	289.04	137.02	1.21
	12	3.35	C_26_H_28_O_14_	[M+H]^+^	Isoschaftoside*	565.16	409.09	2.55
	13	3.35	C_26_H_28_O_14_	[M+H]^+^	Apigenin-8-C-(2”-glucosyl) arabinoside	565.16	457.11	1.53
	14	3.79	C_22_H_24_O_11_	[M-H]^–^	Hesperetin-5-O-glucoside	463.12	301	1.13
	15	3.89	C_27_H_30_O_15_	[M-H]^–^	Kaempferol-3-O-robinobioside (Biorobin)	593.15	285	1.43
	16	3.97	C_24_H_22_O_15_	[M+H]^+^	Quercetin-7-O-(6”-malonyl) glucoside	551.1	303.05	1.06
	17	4.03	C_27_H_30_O_14_	[M+H]^+^	Apigenin-7-O-neohesperidoside (Rhoifolin)	579.17	271.07	1.48
	18	4.04	C_27_H_30_O_14_	[M+H]^+^	Apigenin-7-O-rutinoside (Isorhoifolin)	579.17	271.01	1.33
	19	4.06	C_21_H_20_O_11_	[M+H]^+^	Kaempferol-3-O-glucoside (Astragalin)*	449.11	287.06	2.68
	20	4.07	C_21_H_20_O_11_	[M+H]^+^	Kaempferol-4’-O-glucoside	449.11	287.06	1.26
	21	4.17	C_21_H_20_O_12_	[M-H]^–^	Quercetin-7-O-glucoside*	463.09	301.03	1.14
	22	4.72	C_9_H_10_O_4_	[M-H]^–^	3,4-Dihydroxybenzoic acid Ethyl Ester (Protocatechuic acid ethyl ester)	181.05	108	1.61
	23	5.51	C_9_H_10_O_4_	[M-H]^–^	Ethylparaben	165.06	92.03	1.11
CFP	1	1.19	C_20_H_18_O_14_	[M-H]^–^	4,6-(S)-Hexahydroxydiphenoyl-β-D-glucose	481.06	301	1.62
	2	1.39	C_20_H_18_O_14_	[M-H]^–^	4,6-(S)-Hexahydroxydiphenoyl-D-glucose	481.06	275	2.85
	3	1.51	C_13_H_16_O_10_	[M-H]^–^	6-O-Galloyl-β-D-glucose*	311.07	169.01	3.88
	4	1.51	C_13_H_16_O_10_	[M-H]^–^	3-O-Galloyl-D-glucose*	311.07	169.01	3.13
	5	1.94	C_14_H_20_O_8_	[M-H]^–^	Vanilloloside	315.11	153.02	1.61
	6	1.95	C_13_H_16_O_9_	[M-H]^–^	1-O-Gentisoyl-β-D-glucoside	315.07	153	2.07
	7	2.18	C_20_H_20_O_14_	[M-H]^–^	2,3-Di-O-Galloyl-β-D-Glucose	483.08	169.01	1.03
	8	2.68	C_15_H_18_O_9_	[M-H]^–^	1-O-Caffeoyl-β-D-glucose	341.09	161.02	1.22
	9	2.68	C_15_H_18_O_9_	[M-H]^–^	Vanillic acid-4-O-Glucuronide	341.09	161.02	1.10
	10	2.75	C_20_H_20_O_14_	[M-H]^–^	1,4-Di-O-Galloyl-D-glucose	483.08	169.01	1.43
	11	2.83	C_27_H_30_O_17_	[M+H]^+^	Quercetin-3,7-Di-O-glucoside	627.14	303.05	1.18
	12	3.10	C_7_H_6_O_3_	[M-H]^–^	Protocatechualdehyde	137.02	93.04	1.23
	13	3.10	C_30_H_26_O_12_	[M-H]^–^	Procyanidin B2	577.14	407.08	0.98
	14	3.11	C_7_H_6_O_3_	[M-H]^–^	4-Hydroxybenzoic acid	137.02	93	1.71
	15	3.11	C_13_H_8_O_8_	[M-H]^–^	Brevifolin carboxylic acid	291.01	247	1.67
	16	3.35	C_26_H_28_O_14_	[M+H]^+^	Isoschaftoside*	565.16	409.09	3.05
	17	3.38	C_26_H_28_O_14_	[M+H]^+^	Apigenin-6-C-(2”-glucosyl)arabinoside	565.16	427.1	1.51
	18	3.79	C_22_H_24_O_11_	[M-H]^–^	Hesperetin-5-O-glucoside	463.12	301	1.05
	19	3.89	C_27_H_30_O_15_	[M-H]^–^	Kaempferol-3-O-robinobioside (Biorobin)	593.15	285	1.23
	20	3.97	C_24_H_22_O_15_	[M+H]^+^	Quercetin-7-O-(6”-malonyl) glucoside	551.1	303.05	0.98
	21	4.04	C_27_H_30_O_14_	[M+H]^+^	Apigenin-7-O-rutinoside (Isorhoifolin)	579.17	271.01	1.42
	22	4.06	C_21_H_20_O_11_	[M+H]^+^	Kaempferol-3-O-glucoside (Astragalin)	449.11	287.06	2.15
	23	4.72	C_9_H_10_O_4_	[M-H]^–^	3,4-Dihydroxybenzoic acid Ethyl Ester	181.05	108	1.41

*Indicates the top three compounds from different fractions of CNC with the highest relative content.

### *Camellia nitidissima Chi* inhibited the proliferation of multifarious non-small cell lung cancer cell lines

**FIGURE 3:**
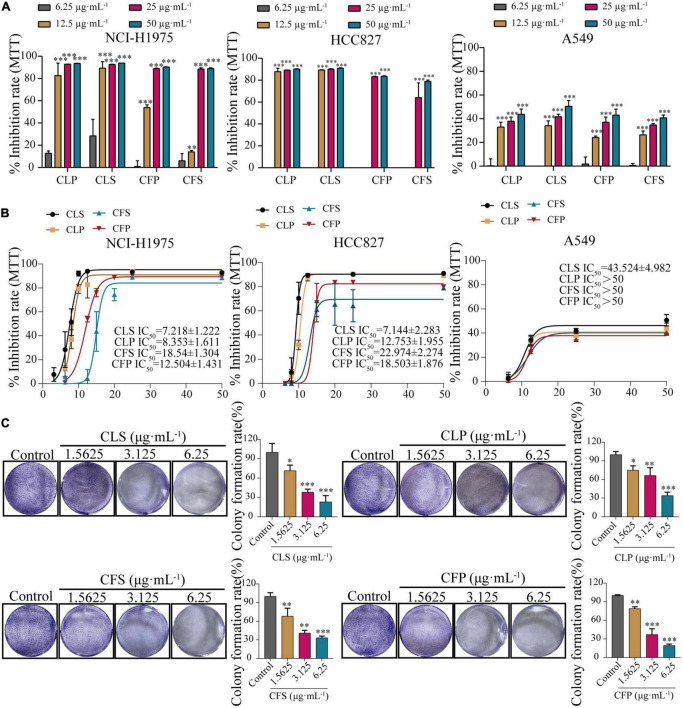
Inhibition of proliferation and clonogenic ability of NSCLC cell lines by four active fractions of CNC. **(A)** Inhibitory effects of different concentrations of CLP, CLS, CFP, and CFS (6.25, 12.5, 25, 50 μg⋅mL^–1^) on NCI-H1975, HCC827 and A549 cells, respectively. **(B)** IC50 of different concentrations of CLP, CLS, CFP, and CFS (6.25, 8, 10, 12.5, 15, 20, 25, and 50 μg⋅mL^–1^) on NCI-H1975, HCC827, and A549 cells, respectively. **(C)** Inhibitory effects of different concentrations of CLS, CLP, CFS, and CFP (1.5625, 3.125, 6.25 μg⋅mL^–1^) on colony formation of NCI-H1975 cells, respectively. *Indicates *p* < 0.05, **indicates *p* < 0.01 and *** indicates *p* < 0.001 relative to the control by ANOVA. The data are presented as the mean ± standard deviation (*n* = 3).

To determine the anti-cancer effect on different non-small cell lung cancer cells, we firstly investigated whether CNC treatment inhibits the proliferation of NSCLC by MTT assay. Treating with CNC significantly suppressed the proliferation of NCI-H1975, A549 and HCC827 cells (Figures 3A,B). After 72 h of treatment, the results confirmed that CLS, CLP, CFS and CFP could significantly inhibit the proliferation of NCI-H1975, A549, and HCC827 cells. It was worth noting that the active fractions of CNC exhibited high inhibitory effect on 3 NSCLC cell lines, especially on EGFR mutant cells NCI-H1975.

Combining the information from the previous MTT assay, we selected NCI-H1975 and HCC827 cells as the main research object. Thus, we performed a colony formation assay by giving CNC every 5 days for 10 days into NCI-H1975 and HCC827 cells. The results demonstrated that CNC treatment significantly restrained anchorage-dependent colony formation of NCI-H1975 and HCC827 cells (Figure 3C and Supplementary Figure 1). At low doses, the NCI-H1975 cells eventually all died as well, demonstrating the remarkable anti-tumor activity of CNC.

**FIGURE 4:**
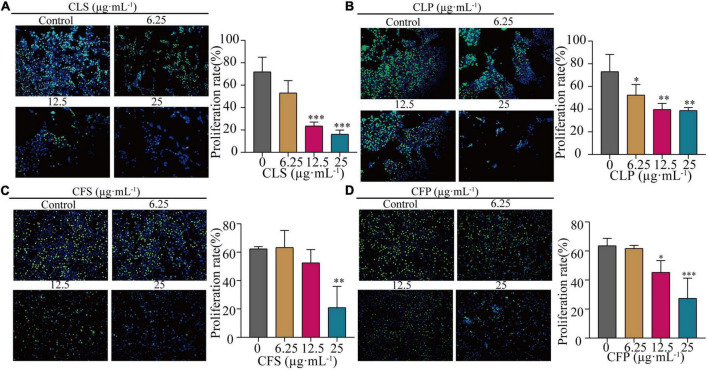
Inhibition of proliferation rate of NCI-H1975 cells by four active fractions of CNC. **(A–D)** Representative field of view of immunofluorescence assay with anti-EdU antibody is shown, where in NCI-H1975 cells, the signal of incorporating with EdU was green and the nucleus was stained blue by Hoechst. Histograms showed the mean values of cell proliferation rates after administration of different concentrations of CLS, CLP, CFS, and CFP (6.25, 12.5, 25 μg⋅mL^–1^). *Indicates *p* < 0.05, **indicates *p* < 0.01 and ***indicates *p* < 0.001 relative to the control by ANOVA. The data are presented as the mean ± standard deviation (*n* = 3).

Furthermore, the EdU assay is one of the most accurate and direct methods for detecting cell proliferation. Observed by laser confocal microscope, the proportion of proliferating NCI-H1975 and HCC827 cells (green) was significantly lower than the control group after 24 h of different concentrations of CNC treatment (Figure 4 and Supplementary Figure 1). Expectedly, CNC treatment led to the significantly decreasing proliferation of NCI-H1975 cells. Simultaneously, we found that CLS had a higher proliferating inhibitory effect on NCI-H1975 cells. These collective data indicated that CNC inhibited the proliferation of NSCLC, supporting that CNC is a new anti-cancer EMP with promising research prospects.

### *Camellia. leave. saponins* induced programmed non-small cell lung cancer death through pyroptosis

**FIGURE 5:**
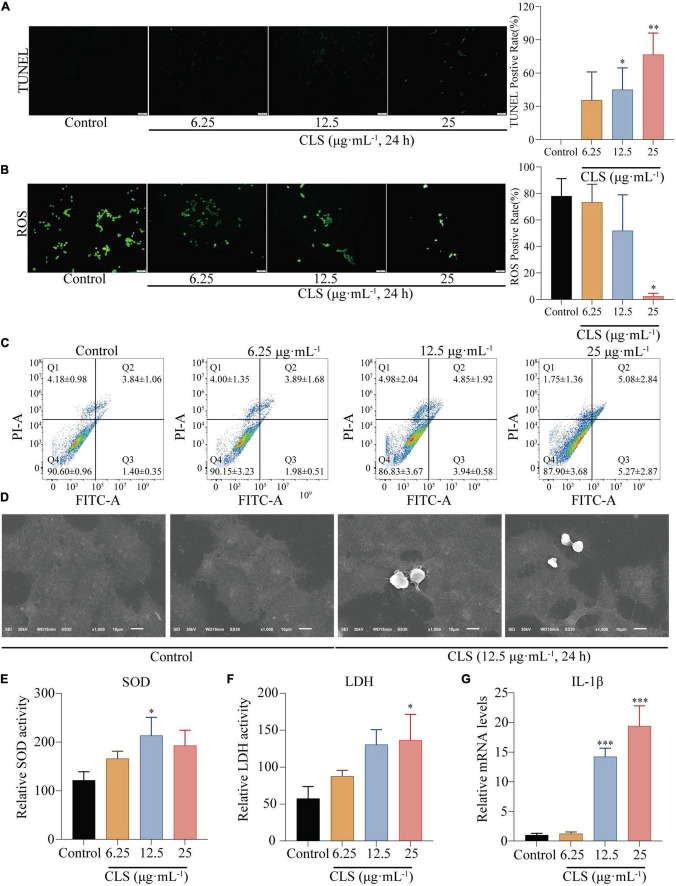
CLS induced programmed NSCLC death through pyroptosis. **(A)** TdT-mediated dUTP Nick-End Labeling (TUNEL) positive rate of NCI-H1975 cells after administration of different concentrations of CLS (6.25, 12.5, 25 μg⋅mL^–1^). **(B)** Reactive oxygen species (ROS) positive rate of NCI-H1975 cells after administration of different concentrations of CLS (6.25, 12.5, 25 μg⋅mL^–1^). **(C)** Annexin V-FITC/propidine iodide (PI) staining of NCI-H1975 cells after 48 h was performed to identify early/late apoptosis, and the data were analyzed *via* flow cytometry. **(D)** SEM was used to detect the morphological changes of NCI-H1975 cells. **(E,F)** Relative uperoxide dismutase (SOD) activity and relative lactate dehydrogenase (LDH) activity of NCI-H1975 cells after administration of different concentrations of CLS (6.25, 12.5, 25 μg⋅mL^–1^). **(G)** Relative IL-1β activity and relative LDH activity of NCI-H1975 cells after administration of different concentrations of CLS (6.25, 12.5, 25 μg⋅mL^–1^). *Indicates *p* < 0.05, **indicates *p* < 0.01 and ***indicates *p* < 0.001 relative to the control by ANOVA. The data are presented as the mean ± standard deviation (*n* = 3).

To determine the programmed cell death effect on non-small cell lung cancer cells, we investigated whether CLS treatment induces the apoptosis of NCI-H1975 cells by TUNEL assay. Treating with CLS significantly induced the apoptosis of NCI-H1975 cells (Figure 5A). Subsequently, we found that CLS inhibited ROS production, suggesting that NCI-H1975 may not induce programmed cell death through ferroptosis (Figure 5B). These results might originate from the antioxidant effect of CNC related (6). Annexin V-FITC/PI assay results showed that CLS treatment significantly unregulated the appearance of labeled cells in Q3 (from 1.98 ± 0.51 to 5.27 ± 2.87) suggesting that there was an increased early apoptosis in NCI-H1975 cells (Figure 5C). Moreover, scanning electron microscope (SEM) showed that the cell in the control group were normal and cell membranes were intact. By contrast, the cell in CLS group showed the damaged cell membranes and evidently increased number of scorched corpuscle (Figure 5D). To further confirm whether the cells underwent pyroptosis, we examined the levels of LDH in the cell supernatant and the relative mRNA expression of IL-1β. These results showed LDH content and IL-1β expression increased with increasing concentrations of CLS administration, which gave the best agreement with CLS induced programmed cell death through pyroptosis (Figure 5E).

### Transcriptome analysis of *Camellia. leave. saponins* -treated NCI-H1975 cells

**FIGURE 6:**
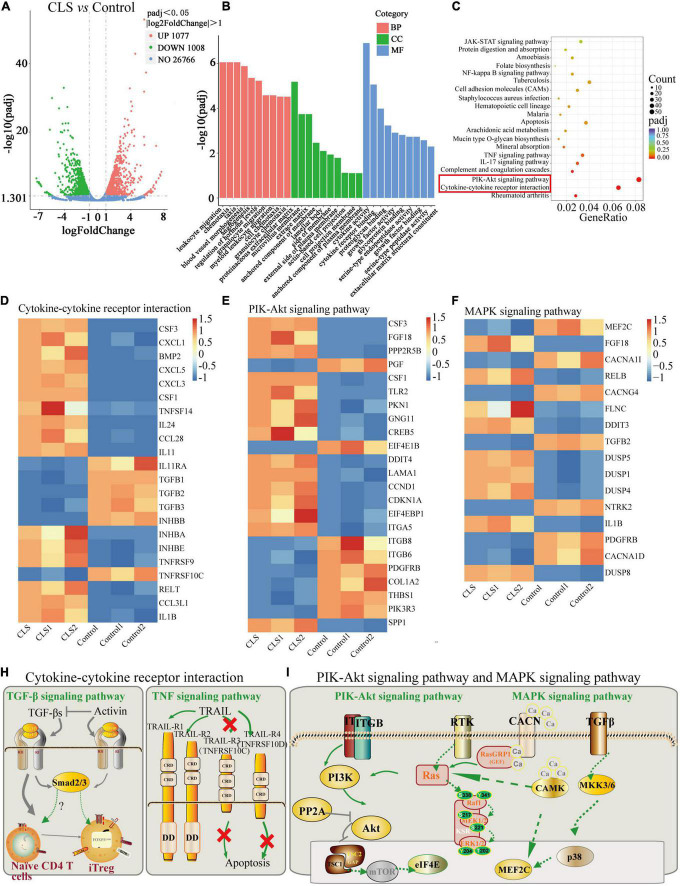
Transcriptome analysis of NCI-H1975 cells between the control group and CLS-treated group. **(A)** Volcano plot with 1,077 DEGs up-regulated and 1,008 DEGs down-regulated. **(B)** GO enrichment analysis of up-regulated and down-regulated DEGs. **(C)** KEGG enrichment analysis of up-regulated and down-regulated DEGs. **(D)** The expression heatmap of 22 DEGs related to Cytokine-cytokine receptor interaction and Rheumatoid arthritis signaling pathway in each sequencing sample. **(E)** The expression heatmap of 23 DEGs related to PIK-Akt signaling pathway in each sequencing sample. **(F)** The expression heatmap of 16 DEGs related to MAPK signaling pathway in each sequencing sample. **(H,I)** Interaction relationships of DEGs on four signaling pathways, including TGF-β signaling pathway, TNF signaling pathway, PIK-Akt signaling pathway and MAPK signaling pathway.

The above studies confirmed the anti-tumor activity of CLS, yet the mechanism of CLS treatment is unknown. Therefore, we applied transcriptome analysis to initially study the mechanism of CLS treatment. The global gene expression changes induced by CLS treatment were determined by comparing the gene profiled NCI-H1975 cells based on microarray data. We found that 1,008 were significantly down-regulated after CLS treatment, while 1,077 genes were significantly up-regulated (Figure 6A), suggesting the global transcriptome changes after CLS treatment.

The pathway-enrichment analysis was annotated based on different databases (GO and KEGG) for homologous alignment to classify the function of differentially expressed genes (DEGs) between control and CLS-treated groups. String-based GO pathway analysis revealed several enriched pathways, including leukocyte migration, chemotaxis, taxis, proteinaceous extracellular matrix, and cytokine activity (Figure 6B). Moreover, “cytokine activity” was the most abundant term for DEGs in the metabolic process which indicated cytokine-mediated signaling pathway could be in-depth investigate (30).

Based on the KEGG, we attempted to perform a standard pathway enrichment analysis to identify the major active pathways for the inhibitory effect of CLS on NCI-H1975 cells. According to the pathway-enrichment analyses of these DEGs (*Q*-value<0.05), the most significantly enriched pathways are “Cytokine-cytokine receptor interaction” and “PI3K-Akt signaling pathway” (Figure 6C). Specifically, 22 DEGs were relevant to “Cytokine-cytokine receptor interaction,” and 23 DEGs of the PI3K-Akt signaling pathway were involved in the anti-tumor process (Figures 6D,E). Transform growth factors (TGF), as an important class of cytokines, have been identified as mediators of a large number of diseases and can regulate the TME (31). TGF can also activate TGFB receptors on the MAPK signaling pathway, thereby affecting the MAPK signaling pathway. Besides, a large number of papers confirmed the existence of multi-level crosstalk between Ras/MAPK and PI3K/Akt signaling pathways (Figure 6H) (32, 33). Moreover, CLS treatment regulated 16 DEGs in the “MAPK signaling pathway” while suppressing NCI-H1975 cells growth (Figure 6F). These results indicated that CLS inhibited NSCLC cells growth *via* multiple targets and pathways, especially by inhibiting signal transduction of “Cytokine-cytokine receptor interaction,” “PI3K-Akt signaling pathway” and “MAPK signaling pathway,” while the involvement of the above pathways needs further experimental verifications (Figures 6H,I).

### *Camellia. leave. saponins* inhibited non-small cell lung cancer *via* multiple pathways and targets

**FIGURE 7:**
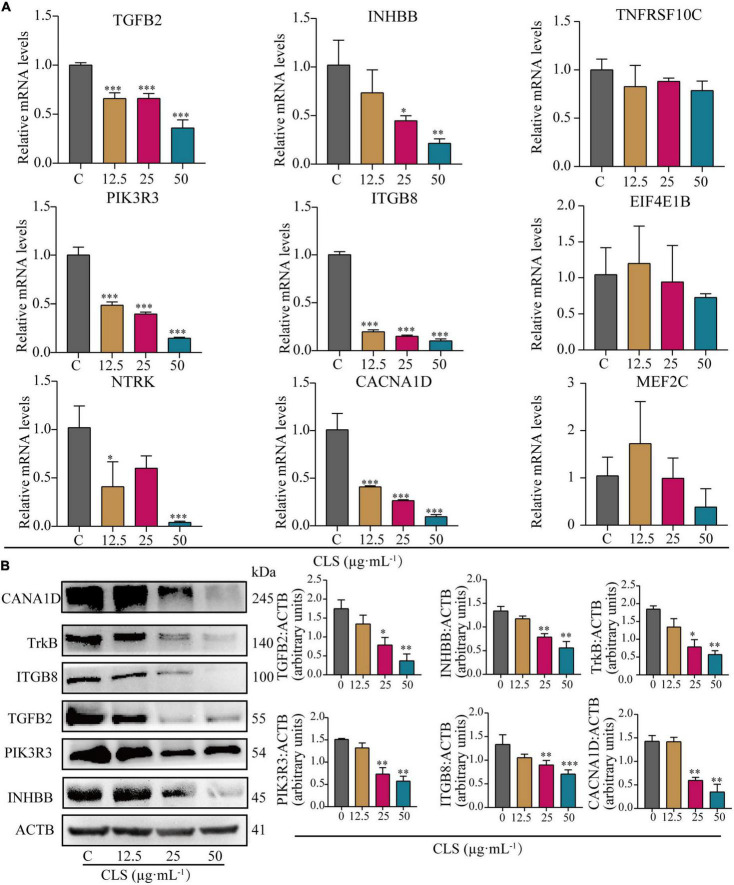
Comparative analysis of quantitative real-time PCR (RT-qPCR) data and Western Blot (WB) data. **(A)** Relative mRNA levels of nine genes including TGFB2, INHBB, TNFRSF10C, PIK3R3, ITGB8, EIF4E1B, NTRK, CACN1D, MEF2C. **(B)** Protein levels of INHBB, PIK3R3, TGFB2, ITGB8, TrkB, CACNA1D detected in CLS-treated NCI-H1975 cells were detected by Western blot with quantification of each protein, *n* = 3 biologically independent embryos. *Indicates *p* < 0.05, **indicates *p* < 0.01 and ***indicates *p* < 0.001 relative to the control by ANOVA. The data are presented as the mean ± standard deviation (*n* = 3).

To verify the results of transcriptome analysis, we used RT-qPCR to validate the key genes of these three pathways separately. As shown in Figure 7A, the results showed that CLS could concentration-dependently inhibit the mRNA levels of TGFB2, INHBB (Cytokine-cytokine receptor interaction), PIK3R3, ITGB8 (PI3K-Akt signaling pathway), NTRK and CACNA1D (MAPK signaling pathway) were relatively significant and concentration-dependent (*p* < 0.05) compared to the control group. The transcriptomic data and RT-qPCR validation unveiled that TGFB2, INHBB, PIK3R3, ITGB8, NTRK (TrkB) and CACNA1D might be a critical targeted gene for NSCLC inhibition by CLS.

To further verify the changes in NSCLC protein expression after CLS treatment, we measured the expression of proteins encoded by crucial genes in NSCLC using the Western Blot assay (Figure 7B). Compared to the control group, the expression of these six key genes was observably decreased. These results were consistent with the result of RT-qPCR, indicating that CLS inhibited Cytokine-cytokine receptor interaction, PIK-Akt and MAPK signaling pathways.

### *Camellia. leave. saponins* suppressed tumor development in nude mice

**FIGURE 8:**
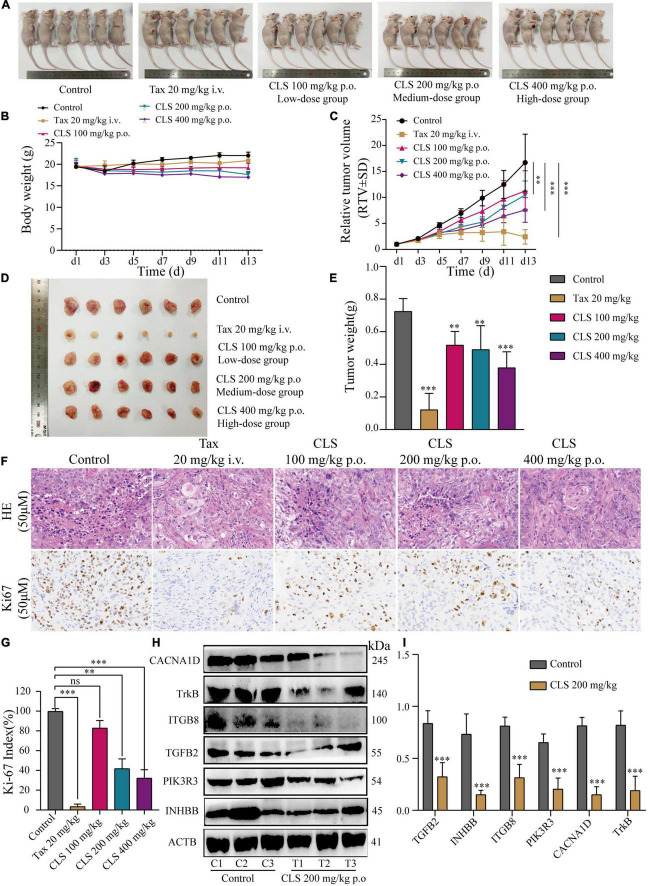
CLS suppressed tumor growth in nude mice. **(A,B)** Signs and weight changes in nude mice, *n* = 6 biologically independent embryos. **(C,D)** Variations in relative tumor volume (RTV) and tumor size in nude mice, *n* = 6 biologically independent embryos. **(E)** Tumor weight in nude mice, *n* = 6 biologically independent embryos. **(F,G)** Morphological observation of tumor by H&E staining and Ki67 detected by DAB yellow staining. **(H,I)** Protein levels of INHBB, PIK3R3, TGFB2, ITGB8, TrkB, CACNA1D detected in CLS-treated tumors were detected by Western blot with quantification of each protein, *n* = 3 biologically independent embryos. *p < 0.05, **p < 0.01, and ***p < 0.001 relative to the control by ANOVA.

To further explore the effect of CLS treatment on tumor development *in vivo*, tumor-bearing mice were treated with different CLS concentrations. NCI-H1975 cells were inoculated subcutaneously into an underarm flank of athymic mice. Intriguingly, the body weight of low-dose, medium-dose and high-dose treated mice were slightly lower than the untreated animals (Figure 8B), but all vital signs such as activity status were normal. In addition, H&E results and physiological and biochemical results of the liver and kidney showed no significant hepatic or renal toxicity with CLS treatment (Supplementary Figure 2). This result might be based on the ability of CNC to inhibit lipase activity (34, 35). Compared with untreated mice in the control group, the CLS 100 mg/kg showed a tumor inhibition effect since day 9 (Figure 8C) while CLS 200 mg/kg and 400 mg/kg treated group significantly suppressed tumor growth starting at day 5 (Figures 8C,D). In addition, the tumor weights of the mice were significantly different in all the administered groups after execution compared to the control group (Figure 8E). Suppression of Ki67 by CLS was also confirmed *in vivo* by IHC analysis in CLS-treated BALB/c-nude mice tumors (Figures 8F,G). Based on the *in vitro* transcriptome analysis and validation of “cytokine-cytokine receptor interaction,” “PI3K-Akt signaling pathway” and “MAPK signaling pathway,” we measured the relative protein expression of TGFB2, INHBB, PIK3R3, ITGB8, NTRK, and CACNA1D in tumor tissue. Interestingly, we found that the paramount targets previously validated by cell samples in Western blot experiments were also corroborated in tumor samples (Figures 8H,I). Taken together, these results proved that CLS treatment could effectively inhibit the growth of NCI-H1975 tumor xenografts in a dose-dependent manner through cytokine-cytokine receptor interaction, PIK-Akt, MAPK signaling pathways.

## Discussion

Although EGFR-TKI have become a first-line inhibitor of EGFR mutation-positive NSCLC, nearly half of NSCLC patients are resistant to EGFR-TKI-based chemotherapies. Thus, it is an urgent need for development of drugs that could inhibit NSCLC with EGFR mutations. In this study, we identified inhibitory effects of different active fractions of CNC on NSCLC cell lines. Four fractions of CNC demonstrated remarkable anti-NSCLC effect. Intriguingly, upon treatment with CLS on NCI-H1975 cells, CLS suppressed the cytokine-cytokine receptor interaction, PIK-Akt and MAPK signaling pathways, leading to growth inhibition of the tumor *in vitro* and *in vivo*. Briefly, this study suggested that CNC, as a functional food, could provide a more efficient treatment in EGFR mutated NSCLC.

As a plant with both medicinal and ornamental value, the pharmacological effects of its flower fractions have been thoroughly studied (34, 36, 37), while the research on leave fractions was rare. Furthermore, few studies have systematically investigated the differences in the chemical composition of the four active fractions of CNC. In this study, we firstly identified the three main components of CLS are isoschaftoside, vicenin-2 and hyperin (Table 3). And recently reported, Vicenin-2 and Hyperin were identified as two novel nature medicine against NSCLC, indicating that CLS possess anti-NSCLC properties and play a crucial role in patients’ defense against tumor (38, 39). However, the pharmacology effects of isoschaftoside have been rarely reported. Hence, further investigation is needed to evaluate the effects of isoschaftoside on NSCLC cells. Besides, the chemical composition of the four active fractions of CNC was found to be various by UPLC-QTOF-MS/MS (Table 4). In the two extracted fractions of the leaves of CNC, the content of geniposide (CLS 1.89 %, CLP 1.68 %) was high, while the content of the two extracted fractions of the flowers was almost absent. Extensive experiments and analysis demonstrate that geniposide possesses relatively strong anti-tumor activity (40, 41) and pulmonary protective effect (42). This suggests that the variation in geniposide content could contribute to the difference in anti-tumor activity between CNC leaf fractions and flower fractions. It is reasonable to make assumptions that the anti-tumor activity of different fractions of CNC also differed based on the composition differences.

Nowadays, few studies have investigated the effect of different active fractions of CNC on NSCLC. In this study, we examined the inhibitory of four active CNC fractions on three cell lines of NSCLC (NCI-H1975, HCC827, A549). These results showed that the four active fractions of CNC possessed remarkable inhibitory on NSCLC cell lines, especially on EGFR-T790-mutated NCI-H1975 cells (Figure 3A). To further confirm the inhibitory effect on EGFR-T790-mutated NCI-H1975 cells, colony formation assay and EdU incorporation assay were performed (Figures 3B, 4). Combining the above results, we concluded that the active fractions of CNC could effectively inhibit the proliferation of NSCLC, among which CLS had the better inhibitory effect on NCI-H1975 (Supplementary Figure 1). In addition, we found that CLS could induced programmed NSCLC death through pyroptosis (Figure 5).

Altered levels of NSCLC-related genes have been inspected by transcriptome analysis after CLS treatment. Interestingly, CLS appears to suppress tumor cell growth *via* “Cytokine-cytokine receptor interaction,” “PI3K-Akt signaling pathway” and “MAPK signaling pathway”(Figure 6). According to transcriptomic results, the expression of TNFRSF10C, INHBB, TGFB1, TGFB2, and TGFB3 were down-regulated after CLS stimulation (Figure 6D). Studies of cytokines suggested that chemokines as a cytokine can promote anti-tumor immunity to NSCLC (43). At present, anti-cytokine antibodies and cytokine blockers have been extensively studied in tumor therapy (44). Transform growth factors (TGF), as an important class of cytokines, have been identified as mediators of a large number of diseases and can regulate the TME (31). Consequently, we choose TGF-β, Activin and TRAIL as the key cytokines and cytokine receptors. In this research, CLS treatment might promote T cell differentiation and tumor immune response by inhibiting the expression of three TGF phenotypes (TGF-β1, TGF-β2, and TGF-β3), thereby inhibiting tumor angiogenesis and invasion. At the same time, by inhibiting TNFRSF10C competitively binding with tumor necrosis factor-associated apoptosis-inducing ligand (TRAIL), TRAIL-induced tumor cell apoptosis can be promoted, thus possibly inhibiting the proliferation and metastasis of tumor cells (Figures 6D,H). Alternatively, tumor cells promote the expression of cytokines to escape the immune response. Overall, the inhibitory of CLS on tumor growth might be exerted by affecting the interaction of cytokines-cytokines receptors. Among them, TGF-β and TNFRSF10C might be the two most critical targets for suppressing NSCLC growth. Therefore, remodeling the immune microenvironment of NSCLC through inhibiting Cytokine-cytokine receptor interaction provides new perspectives for the treatment of NSCLC. Besides, our RT-qPCR and WB results also suggested that the down-regulation of the levels of TGFB2 and INHBB genes might be important targets that suppress the proliferation of NCI-H1975 cells (Figures 7A,B).

TGF-β induces EMT in tumor cells through Smad and non-Smad signaling pathways, whereas non-Smad includes signaling pathways such as PI3K, MAPK (45). Based on transcriptomic data, we found that ITGB8 down-regulation cascades its downstream signal PIK3R3 expression to be suppressed (Figures 6E,I). We hypothesized that the up-regulation of PPP2R5B combined with the down-regulation of PIK3R3 resulted in the inhibition of the Akt-mTOR signaling pathway (Figures 6E,I). As a result, eukaryotic initiation factor 4E (elF4E) was down-regulated. Furthermore, numerous studies have shown that ITGB8 and EIL4E proteins are associated with cancer migration, invasion and metastasis and autophagy (46, 47). We speculate that CLS may induce autophagy-mediated cell death by inhibiting the PIK3R3-Akt-mTOR axis through ITGB8 down-regulation. Mitogen-activated protein kinase (MAPK) signaling pathway, as one of the key pathways to induce tumor production, is involved in a series of cell physiological activities such as cell growth, differentiation and apoptosis (48). According to the transcriptomic analysis, CLS inhibited both CACN receptors (CACNA1D, CACNG4, CACNA1I) and RTK receptors (NTRK2, PDGFRB) and cascade RAS was inhibited (Figures 6F,I). The loss of Ca^2+^ caused by CACN receptors repression also resulted in the down-regulation of RAS and MEF2C expression. It is worth mentioning that intracellular Ca^2+^ can be considered a major regulator of autophagy. Therefore, we selected six genes related to PIK-Akt and MAPK signaling pathways for RT-qPCR validation (PIK3R3, ITGB8, EIF4E1B, TrkB, CACNA1D, and MEF2C), and the results indicated that PIK3R3, ITGB8, NTRK, and CACNA1D could be used as new targets for NCI-H1975 (Figures 7A,B). This observation supports the hypothesis that CLS could induce cell autophagy and inhibit tumor growth *via* PI3K and MAPK signaling pathways. However, the mechanism by which PIK3R3, ITGB8, TrkB, and CACNA1D induce cell autophagy and inhibit tumor growth remains to be elucidated. We speculate that suppression of TGFB2, CACNA1D, TrkB, and ITGB8 could result in reduced PI3K expression, which ultimately would inhibit the metastatic and invasive ability of NCI-H1975 (Figure 6I). In this regard, it has been reported that Vicenin-2 (the content in CLS is 2.36%) inhibited the expression of key proteins of PI3K/Akt and TGF-β/Smad signaling pathway in A549 and NCI-H1299 cells, resulting in reduced EMT (39). These observations suggesting TGFB2, INHBB, PIK3R3, ITGB8, NTRK, and CACNA1D as major mediators in CLS-induced NCI-H1975 cell death. Importantly, these results indicated that CNC as a functional food has the advantage of being multi-channel and multi-target against NSCLC.

Based on the observed effects of CLS on NSCLC, we also constructed the xenograft models assay in nude mice to verify whether CLS is effective *in vivo* NSCLC models. In this work, we found that CLS significantly inhibited the growth of transplanted tumors in nude mice in a concentration-dependent manner (Figure 8A). Interestingly, the slight change in body weight of nude mice with increasing drug doses. However, physiological and biochemical results and H&E sections of the liver and kidney confirmed the absence of significant drug toxicity, suggesting CLS may play a role in lipid-lowering (Figure 8B and Supplementary Figure 2) (35). Also, we performed WB validation in tissue samples of the proteins screened in the previous experiments, with results generally consistent with cell samples (Figures 8H,I). Also, we verified the pathological patterns of tumors and the expression level of Ki67 in tumors by H&E and IHC, which indicating tumor proliferation rate was significantly suppressed (Figures 8F,G). Collectively, our findings identified CLS as a new EMP for NSCLC, providing an essential molecular foundation for enhanced understanding of CNC treatment for NSCLC.

In this work, we preliminarily elucidated the anti-tumor effect by which the four active fractions of CNC against NSCLC and the anti-tumor mechanism of CLS. However, this investigation has several limitations. As an essential method to evaluate pharmacological effects of Chinese medicine, Serum Pharmacology is an important auxiliary analysis method (49, 50). Despite the compositions of different fractions of CNC having been identified, the compounds in serum after oral administration of CLS in mice still require in-depth research. In addition, the specific compounds that affect these signaling pathways and targets still require further corroboration. Although the details of effective compounds and their mechanism in CNC remain unknown, our findings revealed a basic mechanism for the anti-NSCLC effect of CLS, providing scientific support for the application of CNC as a functional food with anti-cancer activity.

## Data availability statement

The original contributions presented in the study are publicly available. This data can be found here: https://www.ncbi.nlm.nih.gov/bioproject/PRJNA891478/.

## Ethics statement

The animal study was reviewed and approved by Hubei University of Chinese Medicine.

## Author contributions

ZW: methodology, data curation, formal analysis, study design, investigation, writing, and validation. XH: study design and writing – original draft. ML: writing – original draft. RJ: software, methodology, and supervision. ZL: software, data curation, and supervision. YW: validation, investigation, and methodology. YG: data curation. DL: formal analysis, software, and supervision. BH: resources, supervision, and writing – original draft. HD: resources, supervision, investigation, writing – original draft and editing, and project administration. All authors contributed to the article and approved the submitted version.

## Supplementary material

Supplementary Figure 1 Inhibition of proliferation and clonogenic ability of HCC827 cells by four active fractions of CNC. **(A,B)** Inhibitory effects of different concentrations of CLS, CLP, CFS, and CFP (6.25 μg⋅mL^–1^) on colony formation and EdU assay of HCCC827 cells. **(C,D)** Inhibitory effects of different concentrations of CLS (1.5625, 3.125, 6.25 μg⋅mL^–1^) on colony formation and EdU assay of HCCC827 cells, respectively. *Indicates *p* < 0.05, **indicates *p* < 0.01 and ***indicates *p* < 0.001 relative to the control by ANOVA. The data are presented as the mean ± standard deviation (*n* = 3).

Supplementary Figure 2 CLS treatment without significant hepatic or renal toxicity. **(A)** H&E pathological sections of liver and kidney tissues. **(B)** Aspartate aminotransferase (AST/GOT) and Alanine aminotransferase (ALT/GTP) of livers in nude mice, *n* = 6 biologically independent embryos. **(C)** Urea nitrogen (BUN) and Sreatinine (Scr) of livers in nude mice, *n* = 6 biologically independent embryos.
