# Supplementary figures and images for: Active fractions of golden-flowered tea (Camellia nitidissima Chi) inhibit epidermal growth factor receptor mutated non-small cell lung cancer via multiple pathways and targets in vitro and in vivo
Source: Front Nutr. 2022 Oct 28;9:1014414. doi: 10.3389/fnut.2022.1014414 (PMC9649924; doi:10.3389/fnut.2022.1014414)

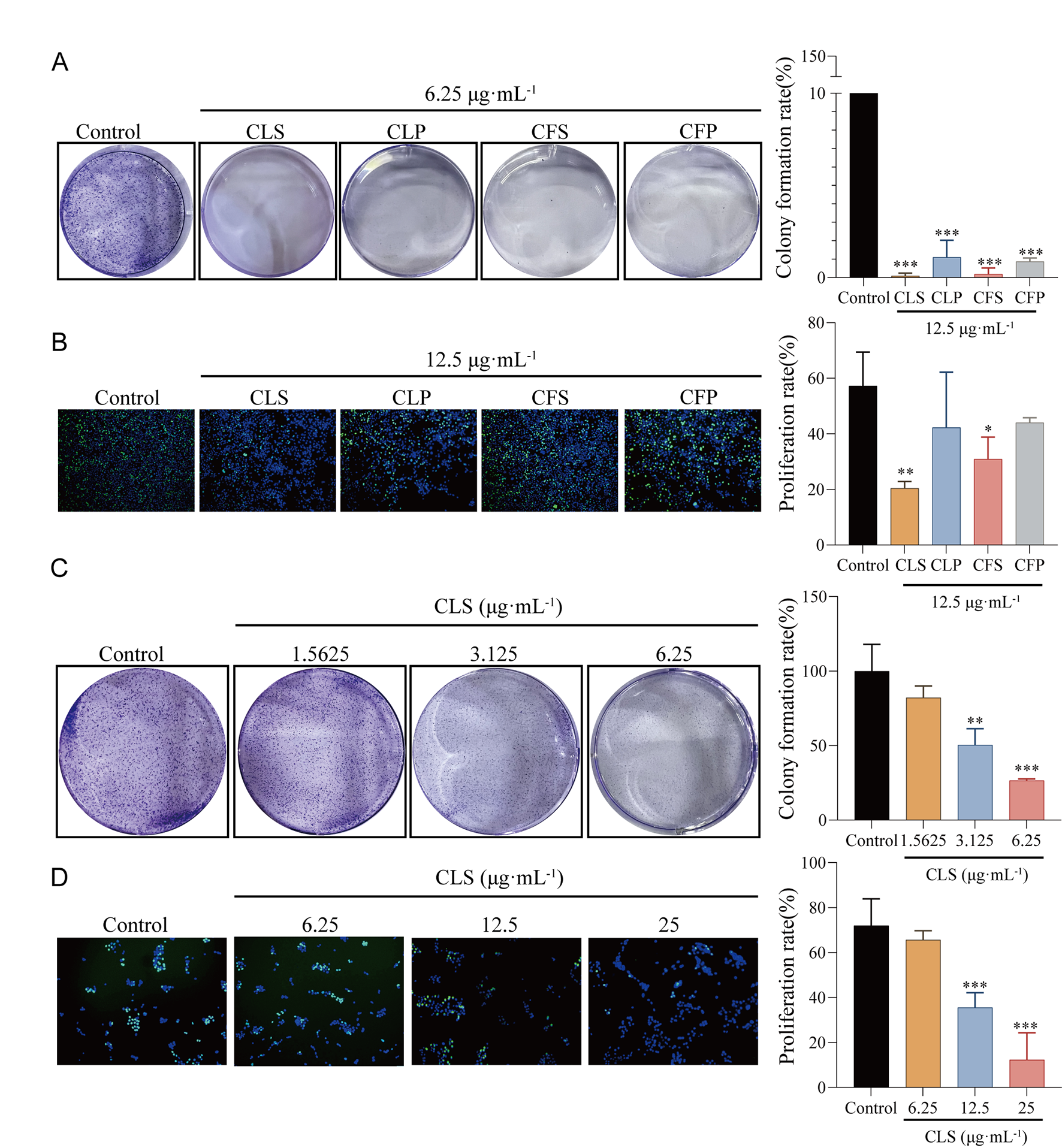

Supplement: Supplementary Figure 1 — Inhibition of proliferation and clonogenic ability of HCC827 cells by four active fractions of CNC. (A,B) Inhibitory effects of different concentrations of CLS, CLP, CFS, and CFP (6.25 μg⋅mL–1) on colony formation and EdU assay of HCCC827 cells. (C,D) Inhibitory effects of different concentrations of CLS (1.5625, 3.125, 6.25 μg⋅mL–1) on colony formation and EdU assay of HCCC827 cells, respectively. *Indicates p < 0.05, **indicates p < 0.01 and ***indicates p < 0.001 relative to the control by ANOVA. The data are presented as the mean ± standard deviation (n = 3). [file Image_1.TIF]

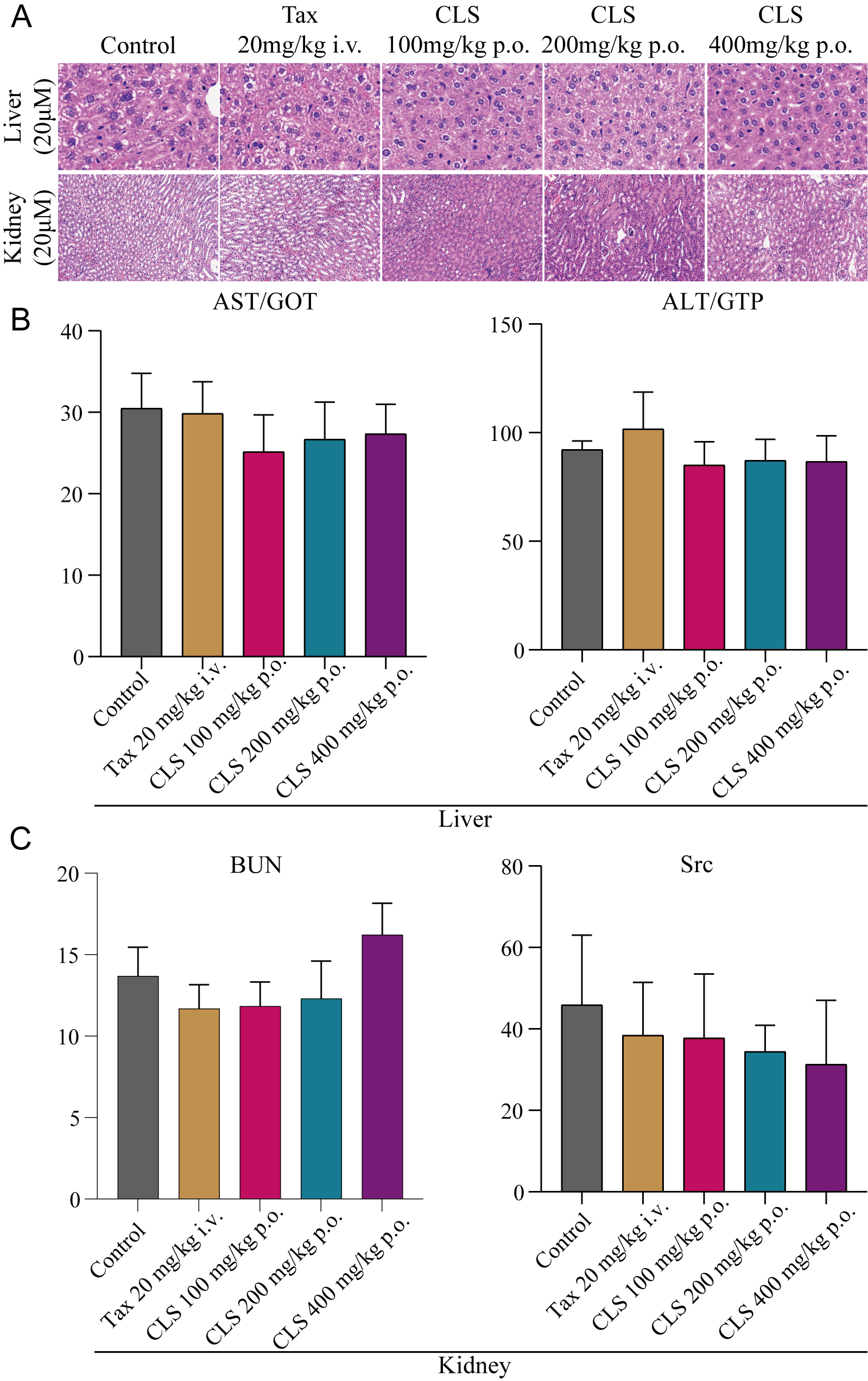

Supplement: Supplementary Figure 2 — CLS treatment without significant hepatic or renal toxicity. (A) H&E pathological sections of liver and kidney tissues. (B) Aspartate aminotransferase (AST/GOT) and Alanine aminotransferase (ALT/GTP) of livers in nude mice, n = 6 biologically independent embryos. (C) Urea nitrogen (BUN) and Sreatinine (Scr) of livers in nude mice, n = 6 biologically independent embryos. [file Image_2.TIF]
